# Prolonged survival of a patient with metastatic leptomeningeal melanoma treated with BRAF inhibition-based therapy: a case report

**DOI:** 10.1186/s12885-015-1391-x

**Published:** 2015-05-13

**Authors:** Dae Won Kim, Edelyn Barcena, Urvi N Mehta, Michelle L Rohlfs, Ashok J Kumar, Marta Penas-Prado, Kevin B Kim

**Affiliations:** 1 Department of Melanoma Medical Oncology, The University of Texas MD Anderson Cancer Center, Houston, TX USA; 2 Department of Radiology, The University of Texas MD Anderson Cancer Center, Houston, TX USA; 3 Department of Neuro-Oncology, The University of Texas MD Anderson Cancer Center, Houston, TX USA; 4 California Pacific Medical Center for Melanoma Research and Treatment, San Francisco Oncology Associates, 2333 Buchanan St., San Francisco, CA 94115 USA

**Keywords:** Metastatic melanoma, Leptomeningeal disease, BRAF inhibitors

## Abstract

**Background:**

Leptomeningeal metastasis of melanoma is a devastating complication with a grave prognosis, and there are no known effective standard treatments. Although selective BRAF inhibitors have demonstrated a significant clinical activity in patients with metastatic melanoma harboring a *BRAF* mutation, the clinical benefit of BRAF inhibitor-based therapy in leptomeningeal disease is not clear.

**Case presentation:**

We present a case of prolonged survival of a patient with *BRAF V600E-*mutant leptomeningeal disease who was treated with vemurafenib followed by whole brain radiation and a combination of dabrafenib and trametinib. Both vemurafenib and the sequential treatment of radiation and dabrafenib/trametinib led to regression of the leptomeningeal disease, and the patient survived for 19 months after the diagnosis of the leptomeningeal disease.

**Conclusion:**

This case suggests a possible clinically meaningful benefit of BRAF inhibitor-based therapy and a need for close investigation of this therapeutic approach in patients with this devastating disease.

## Background

Leptomeningeal disease (LMD) defined as the infiltration of cancer cells in pia mater and arachnoid membrane is a devastating and lethal complication of cancer. It is diagnosed by a combination of suggestive symptoms and signs (often indicating dysfunction of the nervous system at multiple levels), the presence of cancer cells within the cerebrospinal fluid (CSF) and/or demonstration of leptomeningeal enhancement on a magnetic resonance imaging (MRI) scan [1]. Malignant melanoma is one of the most common solid tumors with predilection to leptomeningeal metastasis, occurring in up to 23 % of patients with melanoma [2], and patients with metastatic melanoma involving the leptomeninges have the worst prognosis among all patients with solid tumor-related LMD [3]. The median overall survival of melanoma patients with LMD is only 8–10 weeks [3, 4]. The incidence of LMD has increased over the years [5] and may continue to increase, likely due to the improved overall survival and a prolonged control of extracranial disease with newly approved systemic therapeutic drugs, such as anti-cytotoxic T-lymphocyte antigen (CTLA)-4 antibody and BRAF inhibitors. Unfortunately, there are no known effective therapeutic options for LMD in patients with metastatic melanoma. Therefore, new effective therapeutic modalities are needed for the treatment of LMD.

The rapid technical advances in the molecular and genetic analysis of melanoma have led to the identification of mutations in the *BRAF* gene in melanoma and the development of targeted therapy for *BRAF-*mutant metastatic melanoma. Approximately 50 % of all melanomas contain a kinase-activating *BRAF* mutation at codon 600 of exon 15, a majority of which with a substitution of valine with glutamic acid (*V600E*) [6]. Without treatment with selective BRAF inhibitors, patients with *BRAF V600E*-mutant melanoma generally have a poorer prognosis than those with wild-type *BRAF* [7]. However, selective BRAF inhibitors, such as vemurafenib and dabrafenib, have a significant clinical activity with a clinical response rate of ~50 % and a median progression-free survival duration of nearly 7 months, prompting the approval of these drugs by the Food and Drug Administration (FDA) in the United States [8-11]. Interestingly, a phase II study of dabrafenib demonstrated that it also has a clinically meaningful clinical activity in the brain in patients with metastatic melanoma harboring a *BRAF V600E* mutation, similar to its activity in the extracranial organs [12]. However, the clinical activity of the BRAF inhibitors or BRAF inhibitor-based combination regimens in LMD has not been established yet. Here, we report a patient with *BRAF*-mutant metastatic melanoma who had a great clinical response in the LMD and an unexpectedly long survival with BRAF inhibitor-based treatment.

## Case presentation

**Fig. 1:**
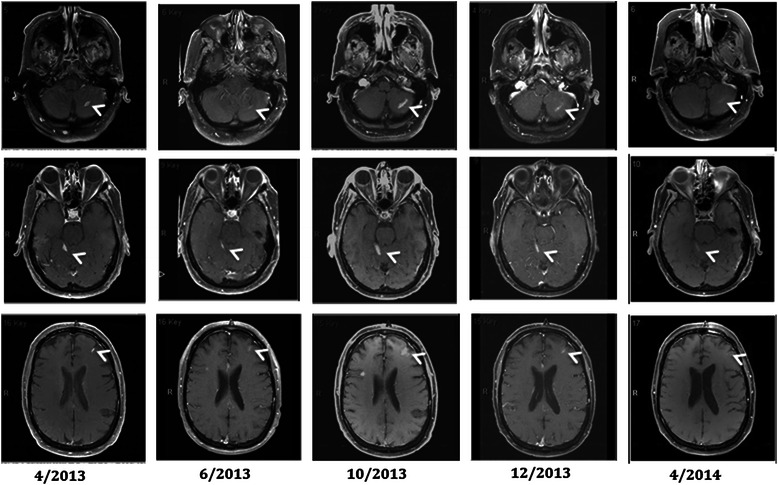
Brain MRI showing the response and the progression of the leptomeningeal disease with BRAF inhibitor-based therapy. The arrows indicate the enhancement of the leptomeninges in April 2013. After vemurafenib therapy, the follow-up images revealed initial regression of the leptomeningeal disease. He had progression of the leptomeningeal disease in October 2013 and started whole brain radiation followed by dabrafenib plus trametinib. The follow-up scans showed regressed leptomeningeal disease

A 61-year-old male, was diagnosed with 5.5 mm thick, Clark level IV, nodular melanoma without ulcerations on his right back in May of 2007. He underwent a wide local excision of the primary melanoma and a sentinel node biopsy in the right axilla, which revealed one lymph node positive for metastatic melanoma. He subsequently underwent a lymph node dissection of the right axilla; none of 32 lymph nodes was positive for metastatic melanoma. After the lymph node dissection, a computed tomography (CT) scan of the body revealed no evidence of metastatic disease. He was fine until September of 2009, when he had a solitary metastatic melanoma in the left lung, for which he underwent left upper lobe lingular-sparing lobectomy with the resection of the nodule in November of 2009. He remained free of disease until November of 2011, when he was found to have new metastatic lesions in the right middle lobe of lung and peritoneum, and he received 4 doses of ipilimumab (3 mg/kg) and then, 2 doses of the combination of TPI-287 (abeotaxane) and temozolomide with further progression of the peritoneal lesions and stable disease in the lung. Subsequently, in June of 2012, the peritoneal lesions were surgically resected. However, in April of 2013, a MRI scan of the brain revealed multiple parenchymal metastatic lesions and clear leptomeningeal enhancement spread to the left cerebellar sulci and the left frontal cortical sulci (Fig. 1). His spinal MRI was normal. He had no neurological symptoms related to the LMD. A CSF analysis demonstrated the presence of few melanoma cells. A CT scan of the body also revealed progression of the lung lesions and new peritoneal masses. He started treatment with vemurafenib at 960 mg twice a day since a molecular analysis showed that his primary melanoma harbored a *BRAF V600E* mutation. He tolerated the treatment well with mild photosensitivity. In June of 2013, a MRI scan of the brain revealed improvement with decrease in size of parenchymal metastatic lesions and regression of leptomeningeal disease (Fig. 1). A CSF examination showed no malignant cells. In addition, a CT scan of the body also demonstrated clinical response in the lung and peritoneal metastatic lesions. Follow-up scans revealed further improvement of LMD in August of 2013. Unfortunately, he had disease progression in the brain, leptomeninges, peritoneum and subcutaneous lesions in October of 2013 (Fig. 1). He was treated with whole brain radiation followed by the combination of dabrafenib and trametinib in November of 2013. He developed moderate fatigue from the treatment, which resolved with decreased dose of dabrafenib and trametinib. A MRI scan of the brain demonstrated improvement of the LMD and brain parenchymal metastatic disease in December of 2013 and February of 2014. As of the most recent follow-up evaluation in April of 2014, a MRI scan of the brain showed continued disease response in all metastatic sites including the leptomeninges and brain parenchyma (Fig. 1). The patient remained free of neurological symptoms throughout the treatment duration. However, his extracranial metastatic disease progressed in the peritoneum and the subcutaneous tissues and he expired in October of 2014 which is 19 months after the diagnosis of brain and leptomeningeal metastases.

## Conclusions

In our report, we presented a case of an unexpectedly prolonged survival in a patient with metastatic melanoma involving the brain and leptomeninges with BRAF inhibitor-based therapy. The clinical response to each of the BRAF inhibitor-based treatments was demonstrated both radiographically and cytologically. The patient tolerated the treatment without development of signs or symptoms suggestive of worsening LMD. Considering that the median overall survival duration of patients with LMD from melanoma is only 8–10 weeks [3, 4], our patient truly had a clinically meaningful benefit from this therapeutic approach.

LMD remains a devastating complication of cancer despite the significant improvement in overall survival of patients with metastatic melanoma with new effective systemic treatments, including selective BRAF inhibitors and anti-CTLA-4 antibody. The impact of these drugs in the clinical outcome of patients with LMD is not known. Typically, for patients with LMD, comfort care or palliative radiation therapy to areas of bulky or symptomatic disease is considered because of the lack of known effective treatment. Although several case studies published over the decades have demonstrated a response or stabilization of LMD with radiation, systemic treatment with temozolomide or ipilimumab, intrathecal interleukin-2 or intrathecal liposomal cytarabine [3, 13–16], these rare successes remain anecdotal, and it is generally accepted that these treatments do not prolong survival.

Recently, two cases of LMD from *BRAF V600E-*mutant melanoma have shown clinical responses to vemurafenib-containing treatment [17, 18]. However, since the two patients were treated not only with vemurafenib but also with other therapies including intrathecal liposomal cytarabine and sequential whole-brain radiation either immediately or 1–3 months prior to vemurafenib, it is not clear whether the responses in the LMD resulted from a pure vemurafenib effect or from the combination with other treatment modalities, such as a late response from the radiation therapy. In contrast to these published cases, our patient initially received only vemurafenib for the treatment of LMD. Although our patient was previously treated with ipilimumab and systemic chemotherapy, the treatment was completed more than 1 year before the patient developed LMD. Therefore, it is unlikely that the response in the leptomeninge is secondary to the late effect of the previous treatments.

Unfortunately, our patient had progression of the LMD and the extracranial lesions after 6 months of the response with single agent vemurafenib treatment, which approximates the median progression free survival duration with vemurafenib treatment [8, 9]. Interestingly, the relapse of the LMD responded to whole brain radiation followed by the dabrafenib plus trametinib treatment.

Trametinib is a selective inhibitor of MEK1/MEK2 which is the only known substrate of BRAF kinase in the mitogen-activated protein kinase (MAPK) pathway. As the reactivation of the MAPK signaling pathway is one of the most important mechanisms of resistance to BRAF inhibitor therapy, a combination of BRAF inhibitor and MEK inhibitor to more completely block the pathway is a rational approach to delay the resistance. Recently, two phase III studies demonstrated the superiority of a combination of dabrafenib and trametinib over a single agent BRAF inhibitor in treatment-naïve patients with advanced melanoma harboring a *V600 BRAF* mutation [19, 20]. However, its clinical benefit in patients whose metastatic melanoma progressed on a prior BRAF inhibitor treatment is only modest at best [21]. In addition, the response in this setting is mostly observed in the extracranial organs, and there are no published data regarding the clinical efficacy of dabrafenib plus trametinib in LMD resistant to a BRAF inhibitor. In our patient, it is interesting to note that the extracranial lesions were not as responsive to the combination of dabrafenib and trametinib as the lesions in the brain and the leptomeninges. Therefore, we can speculate that the radiation therapy to the brain might have enhanced the efficacy of the targeted drugs by either synergizing antitumor activity with the drugs or simply allowing better drug penetration to and accumulation in the CSF [22]. Controlled preclinical and clinical studies will need to be conducted to delineate the mechanisms of action and the therapeutic role of radiation in this combination approach.

The brain, CSF and meningeal membrane are known as sanctuary sites from toxins and drugs due to the presence of the blood-brain barrier [23]. Although several strategies including intrathecal treatment and a combination of systemic therapy and radiation have been used to overcome this barrier and enhance drug delivery in CSF, the clinical outcome of patients with LMD is still poor. One possible explanation for the leptomeningeal response in our patient with vemurafenib is the concurrent presence of multiple parenchymal metastases which might have disrupted the blood-brain barrier and allowed a higher level of vemurafenib in the CSF. Since vemurafenib is much more effective in the *BRAF*-mutated metastatic melanoma lesions than cytotoxic drugs, the clinical effect might have been more noticeable in the leptomeninges. This speculation is consistent with the finding that the blood-brain barrier permeability is increased up to 22 % in and around brain tumor compared with normal brain tissue [24, 25].

The possible drug accumulation in the CSF can also be suggested by the fact that the expression level of a P-glycoprotein, a multidrug resistance protein, which effluxes drugs out of the central nervous system (CNS), is 70–95 % lower in metastatic melanoma than normal brain tissue [25, 26]. In addition, the angiogenesis associated with the progression of melanoma into the leptomeninges results in an abnormal and leaky blood-brain barrier [25, 27], which may further increase vemurafenib or dabrafenib concentration in CSF, though no data regarding the vemurafenib concentrations in the CSF of patients with brain metastases are available. Unfortunately, we could not measure the drug concentration in the CSF for our patient.

In our patient, the LMD was detected by a routine imaging test, and he did not have any symptoms to suggest LMD. The early detection of the LMD in our patient may explain the survival of longer than 1.5 years, since LMD without neurological deficits is associated with good response to treatment [27, 28]. Our speculation is supported by the finding that patients with low tumor burden of metastatic melanoma have a statistically longer median progression-free survival duration with vemurafenib than those with high tumor burden [29, 30]. However, the prolonged survival of our patient is much more remarkable than we expected, since median overall survival duration is only 2.9 months in patients with LMD without neurologic signs or symptoms [31].

The prolonged survival in our patient with BRAF inhibitor-based therapy is very encouraging for the management of patients with melanoma and LMD. Certainly, this approach is not applicable for those with wild-type *BRAF* melanoma, but for those with the *BRAF*-mutant LMD, we hope that our case will spark the interest in well-designed studies to evaluate BRAF inhibitor-based therapy with or without radiation therapy. If this combination therapy is proven effective, it could potentially replace palliative radiation therapy as the preferred therapeutic modality, especially in BRAF inhibitor-naive patients with *BRAF*-mutant melanoma who also have extracranial metastases.

In addition, clinical studies of checkpoint inhibitors, such as anti-CTLA4 antibodies and anti-program death (PD)-1 antibodies, will be needed to improve the disease control in patients with LMD in the future.

## Consent

Written informed consent was obtained from the patient for publication of this case report and any accompanying images.
